# The association between gut microbiota and accelerated aging and frailty: a Mendelian randomization study

**DOI:** 10.1007/s40520-025-02971-3

**Published:** 2025-03-13

**Authors:** Zhiliang Yan, Guoyu Guan, Hanqi Jia, Hanyu Li, Sangdan Zhuoga, Songbai Zheng

**Affiliations:** 1 Department of Gastroenterology, Huadong Hospital, Fudan University, Shanghai, China; 2 Department of Geriatrics, Huadong Hospital, Fudan University, Shanghai, China; 3 Shanxi Medical University School and Hospital of Stomatology, Taiyuan, China

**Keywords:** Mendelian randomization, Gut microbiota, Accelerated aging, Frailty

## Abstract

**Background:**

The recent observational studies have unveiled the correlation between the composition and dynamic alterations of the gut microbiome and aging; however, the causal relationship remains uncertain.

**Aims:**

The objective of this study is to investigate the causal relationship between the gut microbiome and accelerated aging as well as frailty, from a genetic perspective.

**Methods:**

We obtained data on the gut microbiome, intrinsic epigenetic age acceleration, and Frailty Index from published large-scale genome-wide association studies. A two-sample Mendelian randomization analysis was conducted primarily using inverse variance weighting model. We utilized the MR-Egger intercept analysis, IVW method, the Cochran Q test, and the leave-one-out analysis to assess the robustness of the results.

**Results:**

IVW analysis indicated a potential association between Peptococcus (OR: 1.231, 95% CI 1.013–1.497, *P* = 0.037), Dialister (OR: 1.447, 95% CI 1.078–1.941, *P* = 0.014) and Subdoligranulum (OR: 1.538, 95% CI 1.047–2.257, *P* = 0.028) with intrinsic epigenetic age acceleration; while Prevotella 7 (OR: 0.792, 95% CI 0.672–0.935, *P* = 0.006) was associated with a potential protective effect. Allisonella (OR: 1.033, 95% CI 1.005–1.063, *P* = 0.022), Howardella (OR: 1.026, 95% CI 1.002–1.050, *P* = 0.031) and Eubacterium coprostanoligenes (OR: 1.037, 95% CI 1.001–1.073, *P* = 0.042) were associated with an increased risk of frailty; conversely, Flavonifractor (OR: 0.954, 95% CI 0.920–0.990, *P* = 0.012) and Victivallis (OR: 0.984, 95% CI 0.968-1.000, *P* = 0.049) appeared to exhibit a potential protective effect against frailty.

**Conclusion:**

The findings of this study provide further evidence for the genetic correlation between gut microbiota and accelerated aging as well as frailty, enhancing the understanding of the role of gut microbiota in aging-related processes. However, the underlying mechanisms and potential clinical applications require further investigation before any targeted interventions can be developed.

**Supplementary Information:**

The online version contains supplementary material available at 10.1007/s40520-025-02971-3.

## Introduction

Aging is an inevitable and time-dependent complex physiological phenomenon, characterized by a gradual decline in overall bodily functions, thereby emerging as a significant risk factor for various diseases [1]. The occurrence of aging is influenced by a multitude of factors, encompassing genetics, environment, and lifestyle. Dysbiosis of the gut microbiota emerges as a pivotal hallmark of the aging process [2]. Chronic, low-grade, sterile inflammation is believed to underlie the process of aging and age-related diseases, with the gut microbiota playing a central role [3]. The composition of the gut microbiota is established during childhood and remains relatively stable throughout adulthood; however, its assembly, structure and dynamics gradually undergo age-related changes, ultimately resulting in a decline in overall microbial diversity [4]. Data from animal models suggested that dysbiosis of gut microbiota, which is associated with aging, may lead to premature death [5].

Frailty is an age-related clinical condition characterized by a decline in the physiological function of multiple organ systems, which makes individuals more susceptible to stress [6]. The typical manifestations include: weakness, slow gait speed, low physical activity, exhaustion, and unintentional weight loss [7]. The pathophysiological mechanism underlying frailty remains unclear, despite its strong association with adverse events such as falls [8], depression [9], cognitive impairment [10], hospitalization [11], and death [12]. The research conducted by Matt Jackson et al. revealed a negative correlation between frailty and the alpha diversity of the gut microbiome; nevertheless, it failed to establish a causal relationship [13]. Compared to healthy controls, frail older adults exhibit reduced gut microbiome diversity and decreased abundance of bacteria that produce short-chain fatty acids, potentially resulting in heightened permeability of the intestinal mucosal barrier, upregulation of pro-inflammatory cytokines, and the development of sarcopenia [14].

Although observational studies and experiments on animal models have suggested a correlation between aging and gut microbiota, establishing a causal relationship between the two remains challenging. The utilization of Mendelian randomization (MR) studies may present a novel resolution to this predicament. Mendelian randomization is a widely used statistical method based on genome-wide association studies (GWAS) that utilizes genetic variation to simulate randomized controlled trials and infer causal relationships between variables. This analytical approach is more effective in controlling for reverse causality and confounding factors [15, 16]. Our study aims to explore the causal relationship between specific gut microbiota and accelerated aging as well as frailty from a genetic perspective using two-sample MR analysis.

## Methods and design

### Research design

**Fig. 1:**
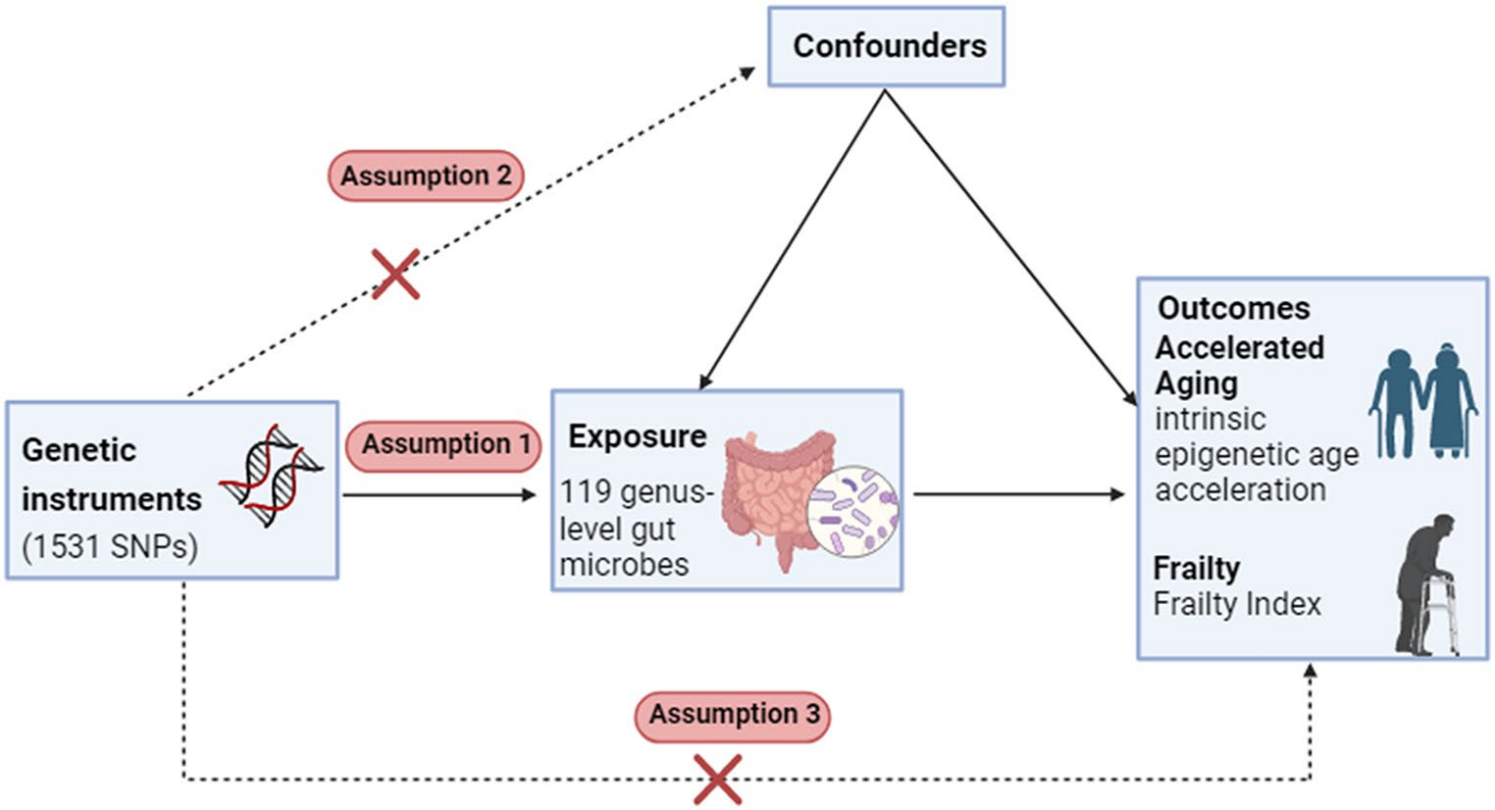
Fundamentals of Mendelian randomization (MR) studies. SNPs, single nucleotide polymorphisms

In this study, we conducted a two-sample MR to estimate the genetic correlation and causal relationships between the gut microbiome and accelerated aging as well as frailty. Mendelian randomization studies employ single nucleotide polymorphisms (SNPs) as instrumental variables (IVs), which are external factors utilized to establish a causal link between an exposure and an outcome. The methodological framework we adopted in our study adheres to three fundamental assumptions, as depicted in Fig. 1: (1) The IVs must exhibit a robust association with the exposure; (2) The IVs are independent of any other potential confounding factors; (3) IVs do not affect the outcome directly, and it can only affect outcome via the exposure.

### Data source

The GWAS data for the gut microbiome were obtained from the MiBioGen consortium, which curated and analyzed genome-wide genotypes and 16 S fecal microbiome data from 18,340 individuals in 24 population cohorts across European (including 16 cohorts with a sample size of 13,266), Hispanic, Middle Eastern, Asian and African ancestries [17].

Recent genome-wide association studies, based on a meta-analysis of 34,710 participants from 28 cohorts of European ancestry, have identified 137 loci associated with DNA biomarkers related to aging [18]. From this study, we obtained summary estimates of the genetic association for intrinsic epigenetic age acceleration (IEAA), which is a derivative of the first generation of epigenetic clock.

Researchers have developed a variety of assessment tools for frailty over the past few decades, but there is still no globally standardized tool. The widely used frailty assessment tools worldwide comprise Frailty Index (FI) [19] and frailty phenotype [20], which have been proven effective in various populations and settings [21]. The frailty index was utilized for the evaluation of frailty in this study. Genetic variants significantly associated with FI were derived from a GWAS meta-analysis conducted on European participants from the United Kingdom Biobank (*n* = 164,610, 60–70 years) and twins from the Swedish TwinGene database (*n* = 10,616, 41–87 years) [22].

### Selection of instrumental variables

The selection criteria for IVs have been established rigorously: (1) The selected SNPs must exhibit a strong association with the exposure, and the filtering criterion for SNP selection is often set at *P* < 5 × 10^− 8^. However, owing to the limited number of SNPs that meet this criterion, we have adjusted the threshold to *P* < 1 × 10^− 5^ based on previous research [23]. (2) To remove genetic linkage disequilibrium, SNPs demonstrating an r^2^ > 0.001 within a 10,000 kb window were excluded [24]. Subsequently, we employed the LDlink website (https://ldlink.nih.gov/) to identify and eliminate SNPs associated with confounding variables linked to the exposure outcomes [25]. (3) SNPs with F statistic > 10 are considered effective IVs that are strongly associated with the exposure, and subsequently, SNPs with a palindrome structure are removed. The information extracted from the database included chromosome, effect allele, other allele, allele frequency (EAF), effect (β), standard error (SE), sample size (N), and *P*-value. In this study, R^2^ = 2 × EAF × (1 - EAF) × β^2^ / (2 × EAF × (1 - EAF) × β^2^ + 2 × EAF × (1 - EAF) × N × SE^2^), F = R^2^ × (*N* − 2) / (1 - R^2^).

### Statistical analysis

The inverse variance weighting (IVW) method is currently regarded as the most precise and reliable approach for conducting MR analysis. We primarily utilized the inverse variance weighting (IVW) method and employed the MR-Egger test and weighted median as supplementary methods to investigate the relationship between exposure and outcomes. The results of the MR analysis were presented as odds ratios (ORs) accompanied by their corresponding 95% confidence intervals (CI).

The reliability of causal effects in this study was ensured through the implementation of a sensitivity analysis. We conducted Cochran’s Q test to assess the heterogeneity of each SNP and generated scatter plots illustrating the associations between SNPs and exposure as well as SNPs and outcomes to visualize MR results. The MR Egger intercept test was used to evaluate the potential horizontal pleiotropy effect. The leave-one-out analysis was conducted to assess the potential impact of each SNP on the outcomes.

All statistical analysis was performed using the “TwoSampleMR” package (version 0.5.11) within the R 4.3.3 statistical software.

## Results

### Instrumental variables

Through meticulous screening, we identified 1531 SNPs that exhibit a significant association with gut microbiota at a level of *P* < 1 × 10^–5^ and an F statistic exceeding 10. The supplemental file1 provides additional details on the instrumental variables.

### MR analysis of gut microbiota and accelerated aging

**Fig. 2:**
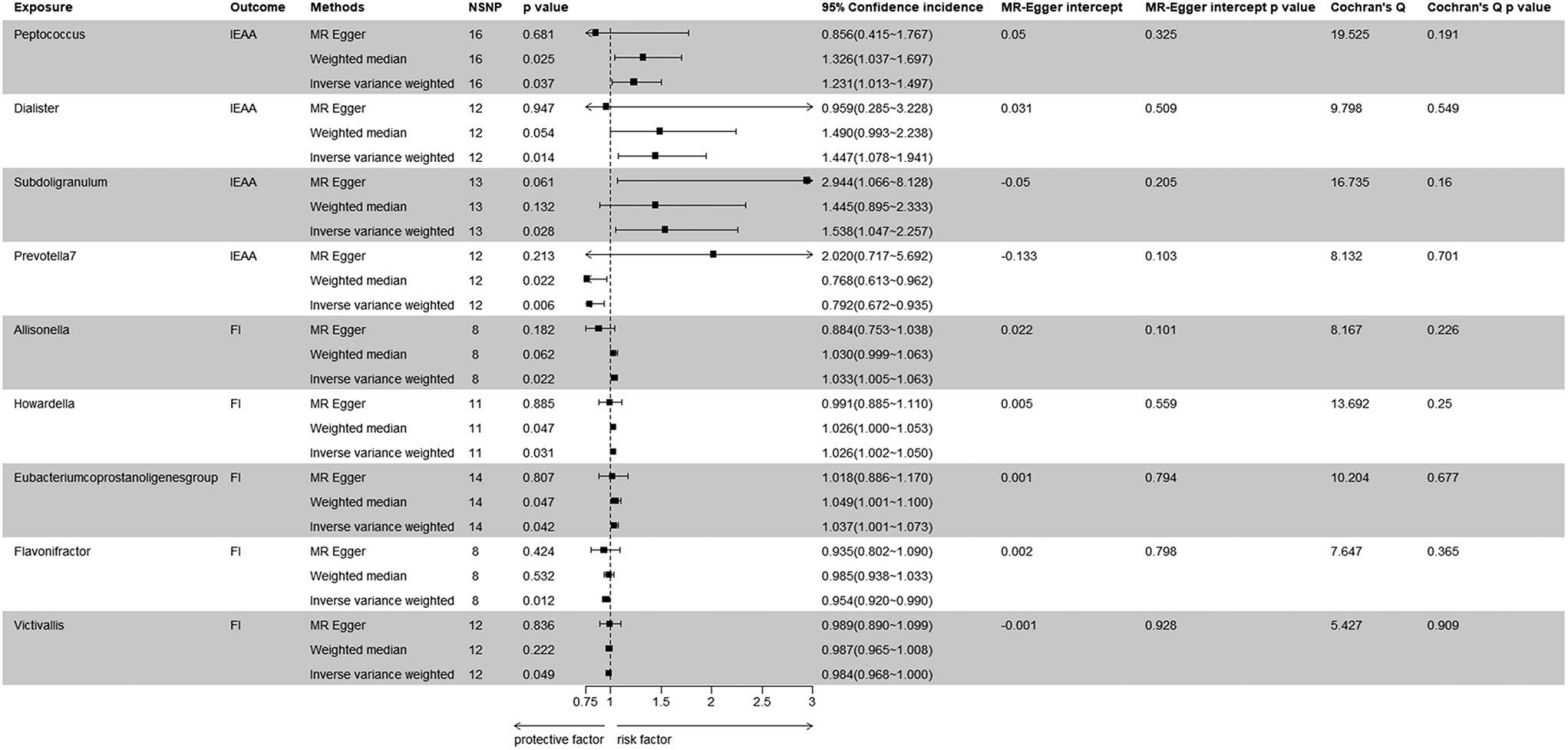
MR results of gut microbiota with IEAA and FI. IEAA, intrinsic epigenetic age acceleration. FI, frailty index

**Fig. 3:**
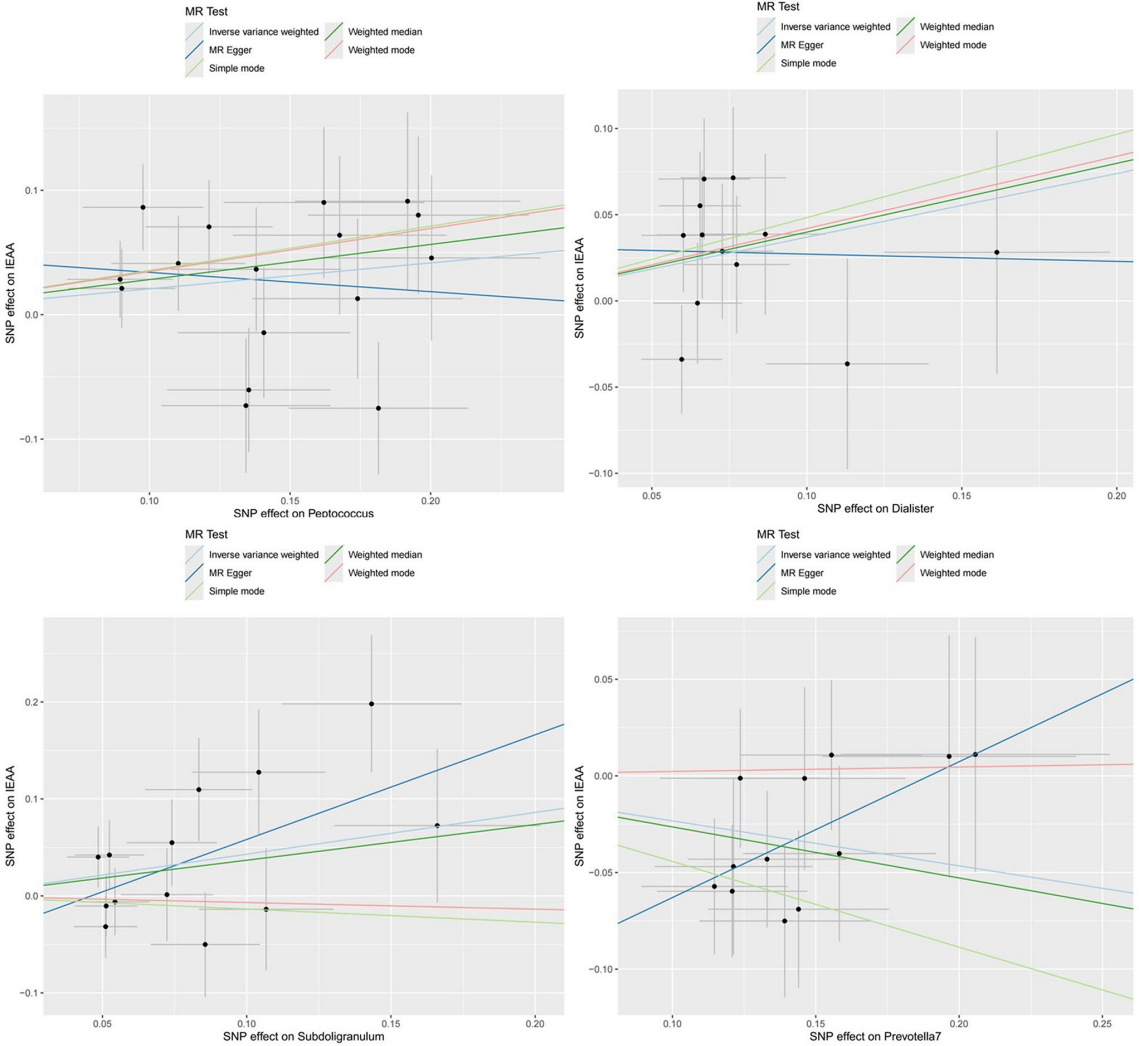
Scatter plots for causal effects of gut microbes on IEAA. IEAA, intrinsic epigenetic age acceleration

As shown in Figs. 2 and 3, the MR analysis suggested that genetic prediction of four microbiotas was associated with accelerated aging. Our primary IVW analysis indicated that Peptococcus (OR: 1.231, 95% CI 1.013–1.497, *P* = 0.037), Dialister (OR: 1.447, 95% CI 1.078–1.941, *P* = 0.014) and Subdoligranulum (OR: 1.538, 95% CI 1.047–2.257, *P* = 0.028) were associated with a higher likelihood of epigenetic age acceleration; conversely, Prevotella 7 (OR: 0.792, 95% CI 0.672–0.935, *P* = 0.006) was associated with a potentially protective effect.

### MR analysis of gut microbiota and frailty

**Fig. 4:**
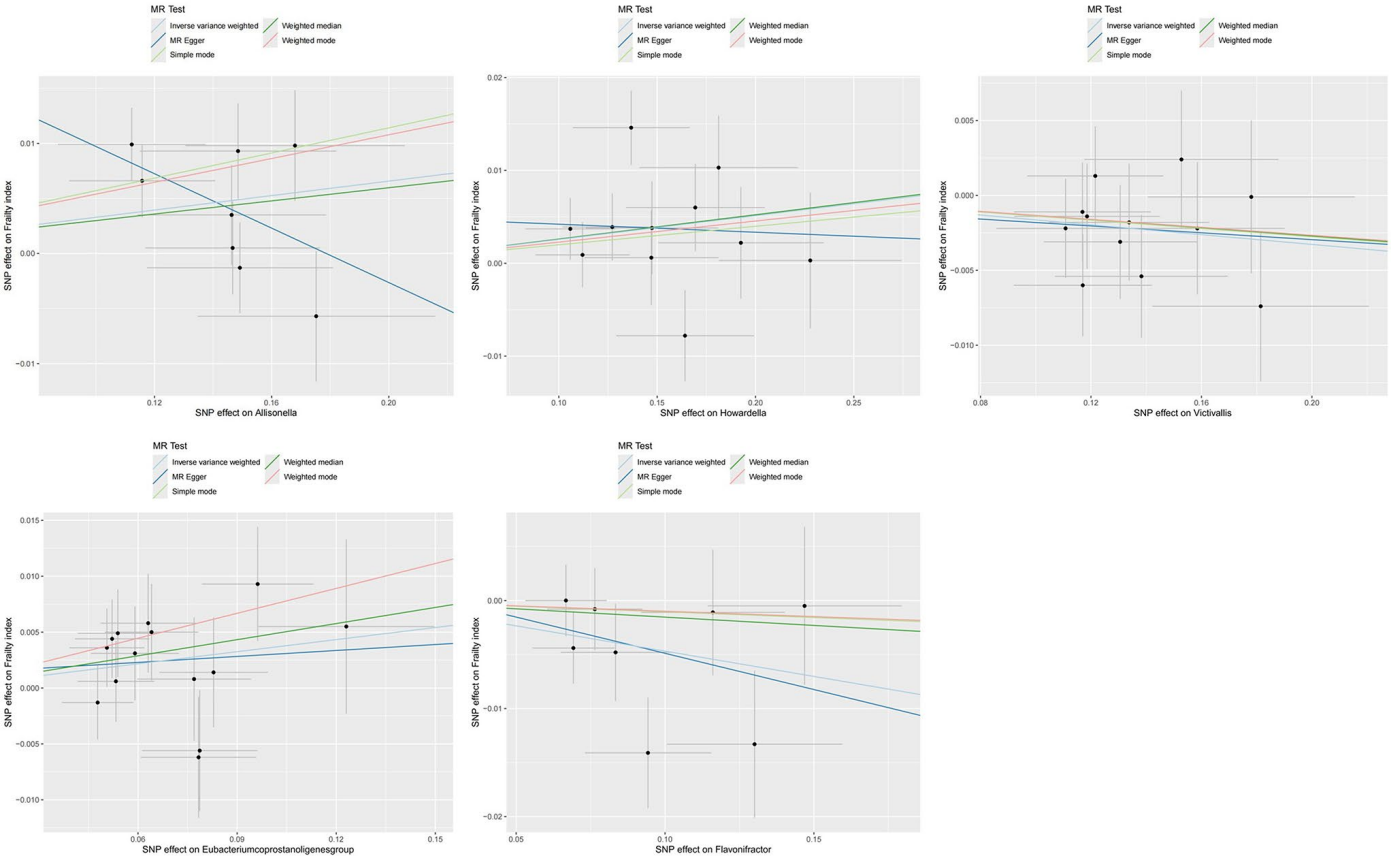
Scatter plots for causal effects of gut microbes on frailty index

Allisonella (OR: 1.033, 95% CI 1.005–1.063, *P* = 0.022), Howardella (OR: 1.026, 95% CI 1.002–1.050, *P* = 0.031) and Eubacterium coprostanoligenes (OR: 1.037, 95% CI 1.001–1.073, *P* = 0.042) were associated with an increased risk of frailty, while Flavonifractor (OR: 0.954, 95% CI 0.920–0.990, *P* = 0.012) and Victivallis (OR: 0.984, 95% CI 0.968-1.000, *P* = 0.049) appeared to exhibit a protective effect against frailty. (Figures 2 and 4)

### Sensitivity analysis

**Fig. 5:**
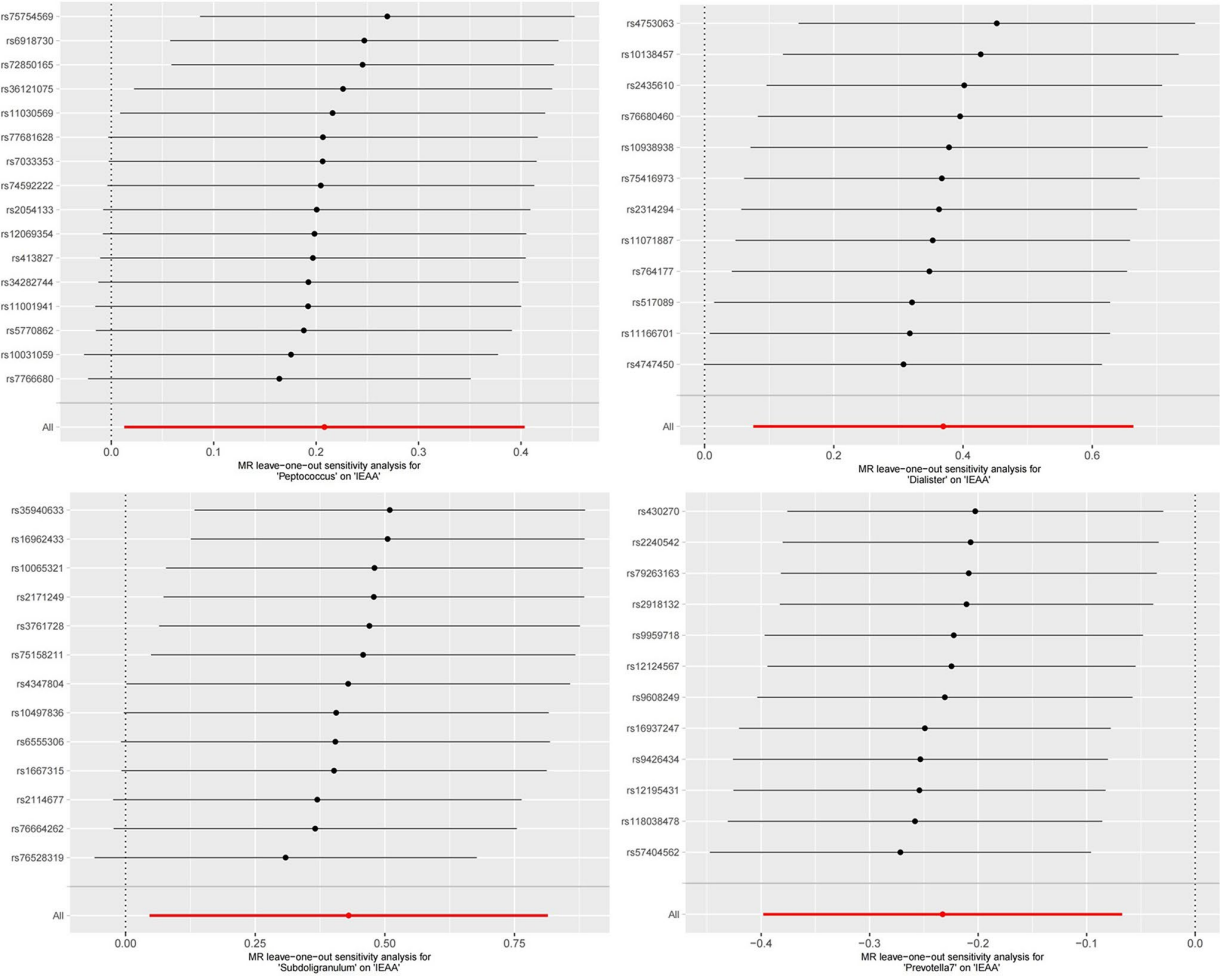
MR leave-one-out sensitivity analysis for gut microbes on IEAA. IEAA, intrinsic epigenetic age acceleration

**Fig. 6:**
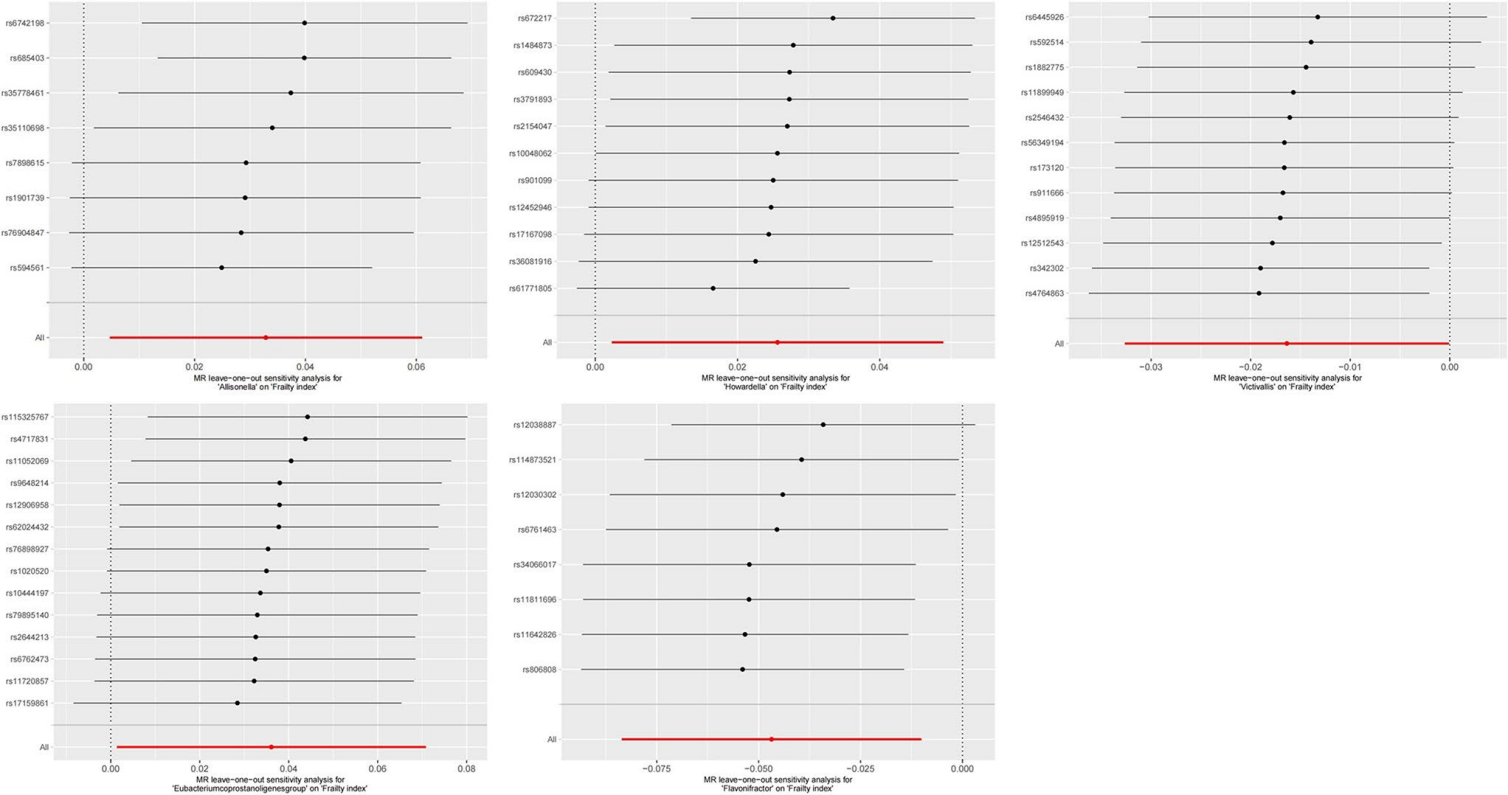
MR leave-one-out sensitivity analysis for gut microbes on frailty index

The MR-Egger regression intercept test did not reveal any indications of horizontal pleiotropy. (Fig. 2) The Cochran’s Q test revealed that all *P* values exceeded the threshold of 0.05, indicating no statistically significant heterogeneity. (Fig. 2) In addition, the leave-one-out analysis further supports the reliability of our results. (Figures 5 and 6)

## Discussion

Emerging evidence suggests a potential association between dysbiosis of the gut microbiome and the aging process as well as age-related diseases; however, further research is required to establish causal relationships and clarify the underlying mechanisms. Identifying reliable biomarkers of aging and developing methods to accurately measure an individual’s biological age have long been key objectives in gerontology. Although chronological age can reflect the degree of aging to some extent, individuals with the same chronological age exhibit varying susceptibilities to age-related diseases and death [26]. The measurement of DNA methylation (DNAm) levels has increasingly become a widely utilized biomarker for assessing biological age and various health-related outcomes in recent years [27]. Consequently, an epigenetic clock has been developed to estimate an individual’s biological age based on DNA methylation levels. An estimated value greater than the chronological age indicates “accelerated aging”, which poses a higher risk of all-cause mortality and multiple adverse health outcomes [28].

This study employed a two-sample MR analysis to investigate the biological impact of specific microbial communities on intrinsic epigenetic age acceleration and frailty. Our study suggested that Peptococcus, Dialister, and Subdoligranulum may be linked to the acceleration of aging processes, while Prevotella 7 exhibits potential protective effects against aging. The gram-negative anaerobic bacterium Prevotella, which was initially described by Shar and Collins in 1990, encompasses diverse species that have been identified and isolated from various anatomical sites including the skin, oral cavity, vagina, and gastrointestinal tract [29]. Sang et al. [30] conducted 16 S rRNA gene sequencing analysis on captive rhesus macaques (Macaca mulatta) and compared this dataset with other freely available gut microbial datasets containing four human populations (Chinese, Japanese, Italian, and British) and two nonhuman primates (wild lemurs and wild chimpanzees). This study identified six common anti-aging gut microbial markers, including Prevotella, which may help produce pivotal metabolites such as butyrate, leucine, and hydroxyproline. There are conflicting reports on the role of Prevotella in human health, particularly in the regulation of glucose metabolism [31, 32]; however, numerous studies have confirmed its association with diet. Francesca et al. [33] found significant associations between consumption of vegetable-based diets and increased levels of faecal short-chain fatty acids, Prevotella and some fiber-degrading Firmicute. Through a systematic analysis of 85 relevant studies, Gabriela et al. [34] found that the abundance of Prevotella within the gut is associated with plant rich diets, abundant in carbohydrates and fibers. These discoveries also align with the traditional dietary pattern for promoting healthy aging, which suggests that a diet centered on plant-based foods improves health outcomes in multiple aspects, including cognitive, psychological, and sensory functions [35]. Dialister is widely recognized for its association with oral infectious diseases [36], and its involvement in expenditure and metabolism has been progressively elucidated. According to Zhang et al. [37], Dialister may serve as a biomarker for predicting obesity, while David et al. [38] found that an increased abundance of Dialister was associated with a weight loss of less than 5% through comprehensive lifestyle intervention. Moreover, numerous studies have demonstrated a higher abundance of Peptococcus in individuals afflicted with obesity and obesity-related diseases [39, 40]. The pathophysiology associated with obesity shares similarities with that observed in normal aging, encompassing alterations in metabolic regulation, insulin resistance, inflammation, and compromised immune function. Substantial evidence suggests the potential for obesity to accelerate the aging process [41]. Limited research has been conducted on Subdoligranulum and its association with aging; however, some studies have indicated a higher abundance of this bacterium in individuals aged over 40 years, which is correlated with impaired metabolism and chronic inflammation [42].

This study suggested Allisonella, Howardella, and the Eubacterium coprostanoligenes may be associated with an increased risk for frailty, while the Flavonifractor and Victivallis may have protective effect. Inflammation, serving as a driving force behind intestinal permeability and dysbiosis, constitutes a pivotal cause contributing to the process of aging and age-related conditions [43]. Paula et al. [44] conducted an analysis on the gut microbiome of patients with different states of inflammation, revealing a higher abundance of the Allisonella genus in subjects with a high inflammation index, thereby suggesting its potential contribution to frailty through pro-inflammatory mechanisms. Although the underlying mechanisms of frailty remain incompletely understood, extensive epidemiological studies have shown that age, prediabetes, diabetes, and low total cholesterol are all associated with an increased risk of frailty [45]. Yang et al. [46] confirmed that Howardella genus was highly abundant in the gut of prediabetic patients; while Douglas et al.’s research suggested that Eubacterium coprostanoligenes was involved in cholesterol metabolism and associated with a reduction in serum total cholesterol [47]. These pieces of evidence suggest that Howardella and Eubacterium coprostanoligenes genera may contribute to the underlying mechanisms governing glucose and lipid metabolism, thereby implicating their involvement in frailty. On the contrary, the abundance of Flavonifractor is negatively correlated with obesity [48], potentially exerting regulatory effects on inflammatory responses by producing butyrate [49, 50], thereby reducing vulnerability to stressors, maintaining functional reserves, and delaying the onset of frailty [51]. Victivallis is also one of the producers of short-chain fatty acids [52] and has been linked to a reduction in obesity and hepatic steatosis in mice [53].

The advantage of our study lies in the utilization of two-sample MR to investigate the association between gut microbiota and accelerated aging and frailty, thereby partially mitigating confounding factors and reverse causality. The reliability of the results was further confirmed through sensitivity analysis. The intrinsic epigenetic age acceleration based on DNA methylation level was chosen as a biomarker of accelerated aging, providing a robust approach to quantify biological aging.

To be transparent, our study has certain limitations. Firstly, the data on intrinsic epigenetic age acceleration and gut microbiota used in this study predominantly come from European populations. Given the potential differences in microbiota composition and genetic backgrounds across ethnic groups, the findings may not be fully generalizable to non-European populations. Secondly, the frailty index in this study combines clinical and functional indicators. While it provides a broad assessment, its subjectivity and exclusion of some factors may lead to biased or incomplete frailty evaluation, potentially resulting in biased conclusions. Finally, the gut microbiome is influenced by various factors such as diet, medications, and environmental conditions. Its dynamic nature can introduce variability, potentially impacting the stability and reproducibility of the results.

## Conclusion

This study provides evidence suggesting the potential influence of gut microbiota on accelerated aging and frailty, as demonstrated through two-sample MR analysis. These findings lay the groundwork for targeted microbiota-based interventions in the field of aging. The elucidation of underlying mechanisms and the exploration of potential clinical applications require further investigation.

## Electronic supplementary material

Below is the link to the electronic supplementary material.

Supplementary Material 1

Supplementary Material 2

Supplementary Material 3

Supplementary Material 4

Supplementary Material 5

## Data Availability

Data is provided within the manuscript and supplementary files.
