# Supplementary material for: The association between gut microbiota and accelerated aging and frailty: a Mendelian randomization study
Source: Aging Clin Exp Res. 2025 Mar 13;37(1):82. doi: 10.1007/s40520-025-02971-3 (PMC11903541; doi:10.1007/s40520-025-02971-3)
Supplement: Supplementary file 3 — Supplementary Material 3 [file 40520_2025_2971_MOESM3_ESM.pdf]

Large-scale association analyses identify host factors influencing human gut microbiome composition

Supplementary Notes and Figures

Supplementary Note 1. Cohort Descriptions

BSPSPC (PopGen)

The PopGen cohort (mean age 61.5 (16.6), 55% male) is a population-based cohort from the area around Kiel, Schleswig-Holstein, Germany. Participants were genotyped using the Affymetrix Genome-Wide Human SNP Array 6.0. Fecal samples of 714 individuals were collected by the participants themselves at home in standard fecal collection tubes and shipped to the study center, where they were stored at -80°C until processing. DNA from fecal samples (app. 200 mg) was extracted using the QIAamp DNA stool mini kit automated on the QIAcube. Genotyping data generation, extraction of fecal DNA and sequencing of the V1-V2 variable region of the 16S rRNA gene and all data processing were performed at the Institute of Clinical Molecular Biology, Kiel, Germany. The study was approved by the institutional ethical review committee of Kiel University, Germany. Written informed consent was obtained from all participants.

CARDIA (Coronary Artery Risk Development in Young Adults Study)

Coronary Artery Risk Development in Young Adults Study (CARDIA) is a population-based prospective study of the evolution of cardiometabolic disease. African American and European American adults were recruited from four U.S. urban areas (Birmingham, AL; Chicago, IL; Minneapolis, MN; Oakland, CA in 1985-1986) (n=5,115, aged 18-30). They have subsequently been examined nine times. A microbiome study was initiated at the Year 30 follow-up examination (2015-2016) in a subset of participants (n=615) who had not taken antibiotics in the past month. Fecal DNA was extracted with the MoBio PowerSoil kit, and the V3-V4 region of the 16S rRNA gene was sequenced with Illumina MiSeq (2x300bp) at HudsonAlpha Institute for Biotechnology (Huntsville, AL, USA). A subset of cohort participants has been genotyped with

the Affymetrix Genome-Wide Human SNP Array 6.0. After quality control and removal of participants with non-overlapping data on microbiome and host genetics, data from 114 African Americans and 257 European Americans (total n=371) were available for analysis.

The study was approved by Institutional Review Boards of University of Alabama at Birmingham, Birmingham, AL, Kaiser Permanente Division of Research, Oakland CA, University of Minnesota, Minneapolis, MN, and Northwestern University, Chicago, IL. Written informed consent was obtained from all participants.

COPSAC$_{2010}$

The Copenhagen Prospective Studies on Asthma in Childhood 2010 (COPSAC$_{2010}$) cohort is a prospective mother-child cohort of 700 children and their families, recruited during week 24 of pregnancy, with written informed consent obtained from all mothers[82]. The participants reside in and around Copenhagen, Denmark. The design builds upon the previous COPSAC$_{2000}$ cohort[83] and is based on detailed longitudinal clinical assessments of asthma, allergy, eczema and other outcomes. Blood tests were taken from the infants at age of six months, and DNA was extracted from plasma. Genome-wide genotyping was performed using the Illumina OmniExpress-8 v1.4 and Exome BeadChip. Fecal samples were collected at visits to the clinic or at home by parents using detailed instructions at ages 1 week, 1 month, and 1, 4, 5, and 6 years. For the present study, samples for age 4-6 years were used. Genomic DNA was extracted from the child's samples using the PowerMag® Soil DNA Isolation Kit, and the V4 region of the 16S rRNA gene was amplified and sequenced on an Illumina MiSeq system, as previously described in detail[84]. At the relevant timepoint, we had both genotype and microbiome data for 380 children to include in this study, 73 of whom had taken antibiotics in the six months before the fecal sample date. The study was approved by Danish Ethics Committee (H-B-2008-093) and the Danish Data Protection Agency (2008-41-2599).

DanFunD (The Danish study of Functional Disorders)

DanFunD is a population-based cohort initiated to outline the epidemiology of functional somatic syndromes[85]. The study population comprises a random sample of 9,656 men and women aged 18-76 years from the general population who were examined from 2011 to 2015. Genotyping using the Human OmniExpress Bead Array (Illumina Inc., San Diego, CA, USA) was conducted on human leukocyte DNA for the entire cohort. A subset of 2,464 participants

volunteered to provide a fecal sample collected under standardized conditions. Microbial DNA extraction using the NucleoSpin Soil kit (Macherey-Nagel, Düren, Germany) and subsequent sequencing of the hypervariable region V4 of the bacterial 16S rRNA gene on an Illumina HiSeq 2500 platform was conducted at Beijing Genomics Institute (BGI Europe, Copenhagen, Denmark). In total, 2,396 samples passed the QC for genotyping and 16S sequencing and were included in the GWAS.

The study was approved by Ethical Committee of Copenhagen County (Ethics Committee: KA-2006-0011; H-3-2011-081; H-3-2012-0015) and the Danish Data Protection Agency. Written informed consent was obtained from all participants.

FGFP (Flemish Gut Flora Project)

The FGFP is a population-based study cohort of 2,482 individuals from the Flanders region of Belgium. Blood and stool samples of volunteers were collected between June 2013 and April 2016. Genotyping was performed using the Human Core Exome arrays v1.0 and v1.1. Sampling kits were sent to the volunteer's homes and stored there at -18°C until collection and storage in the Raes Lab facilities at -80°C. DNA was extracted from the frozen fecal samples using the PowerMicrobiome RNA Isolation Kit, as described in Falony et al[3]. Sequencing of the V4 region of the16S rRNA gene was carried out on the Illumina HiSeq platform. After quality control, 2,259 samples had genotype and 16S data (1,328 females, 896 males, mean age 52.3 yrs). FGFP procedures were approved by the medical ethics committee of the University of Brussels–Brussels University Hospital (approval 143201215505, 5/12/2012). A declaration concerning the FGFP's privacy policy was submitted to the Belgian Commission for the Protection of Privacy. Written informed consent was obtained from all participants.

FOCUS

The FoCus cohort (mean age 51.4(14.6) yrs, 42% male) is a population-based cohort from the area around Kiel, Schleswig-Holstein, Germany, and part of the competence network Food Chain Plus (FoCus, http://www.focus.uni-kiel.de/component/content/article/88.html). Participants were genotyped using the Infinium OmniExpressExome Array. All data generation and processing was performed at the Institute of Clinical Molecular Biology, Kiel, Germany, similar to the PopGen cohort.

The study was approved by the institutional ethical review committee of Kiel University. Written informed consent was obtained from all participants.

GEM (The CCC GEM project)

The CCC GEM project is a prospective international research study designed to identify the potential triggers that contribute to the onset of Crohn's Disease. Since 2008, the GEM project has recruited over 5,000 healthy first-degree relatives of Crohn's Disease patients with an age range of 6-35 years. At the time of recruitment, participants were screened using a standardized questionnaire to exclude any history or symptoms of inflammatory bowel disease or other gastrointestinal diseases. For the microbiome GWAS, we used data from participants recruited in Canada (n=1,115), the United States (n=17) and Israel (n=111). Stool DNA was extracted using the QIAamp DNA Stool Mini Kit (Qiagen, Hilden, Germany). The V4 hypervariable region of bacterial 16S ribosomal RNA (16S rRNA) was sequenced using a MiSeq platform (Illumina Inc. San Diego, CA, USA) and primers 515F/806R[86]. Genotyping of the cohort was performed using the HumanCoreEXOME-12v1.1 chip (n=379), HumanCoreEXOME-24v1.0 chip (n=203) and both ImmunoChip and HumanCoreEXOME-12v1.1 chip (n=662) (Illumina, Inc. San Diego, CA, USA). Thus, in mbQTL mapping, the cohort was split into subcohorts GEM_v12, GEM_v24 and GEM_ICHIP respectively. Among subcohorts, GEM_v24 mostly comprises individuals of Israeli ethnicity (70%, 61 Ashkenazi, 34 Sephardic, 18 other/unknown subethnicities), while the other two subcohorts are of a European ancestry. Only one sample from one member from each family enrolled in the project was included in the current microbiome GWAS study. After stringent QC, as previously described[9], the overlap between samples with genotyping and microbial 16S sequencing data yielded 1,243 samples (676 females, 567 males, median age 19.0(8.03) yrs) for use in the microbiome GWAS analysis. None had used antibiotics in the three months before fecal collection.

The study was approved by Mount Sinai Hospital Research Ethics Board (Toronto–Managing Center) and local centers. Written informed consent was obtained from all participants.

GenR (The Generation R Study)

GenR is a population-based, prospective, multi-ethnic pregnancy cohort study from fetal life until young adulthood. It is conducted in the city of Rotterdam, the Netherlands[87]. Genotyping of this cohort was performed using Illumina HumanHap 610K[88]. Stool sample collection started in

2012 and comprised 2,111 children. Fecal DNA was extracted using DiaSorin Arrow DNA (Isogen Life Science, De Meern, the Netherlands) with a bead-beating step. Sequencing of bacterial 16S gene, domain V3-V4, was performed in the Laboratory of Human Genetics at Erasmus MC Rotterdam using the Illumina MiSeq platform[89]. After stringent QC, the overlap between samples with genotyping and microbial 16S sequencing data yielded 1,328 samples (656 females, 672 males, mean age 9.8(0.3) years) for use in the microbiome GWAS analysis. None had used antibiotics in the six months before fecal collection.

The study was approved by the Medical Ethical Committee of Erasmus MC, University Medical Center Rotterdam. Written informed consent was obtained from all participants.

KSCS (Kangbuk Samsung Cohort Study)

The KSCS is a prospective cohort study to evaluate the natural history, prognosis and genetic and environmental determinants of a wide range of health traits and diseases among Korean adults. There are two major cohort studies in Kangbuk Samsung Hospital: the KSCS and the Kangbuk Samsung Health Study (KSHS). The KSHS is a retrospective cohort study using de-identified data routinely collected during health screening visits from 2002 to present, and includes standardized and high quality clinical, imaging and laboratory procedures and information on multiple lifestyle and medical conditions. The KSCS is a prospective study that has now started to apply more strict and standardized procedures. They obtained informed consent for data linkage to national registries for death, cancer and medical utilization since 2011. Genotyping was conducted using the Illumina HumanCore BeadChips 12v in 2014 (n=2,040). Fecal samples were collected from 1,463 participants between June and September 2014. DNA extraction from fecal samples was performed within one month of storage using the MoBio PowerSoil ® DNA Isolation Kit (MO BIO Laboratories, Carlsbad, CA, USA). Sequencing of the bacterial 16S rRNA gene, domain V3-V4, was performed using the Illumina MiSeq platform (Illumina, San Diego, CA, USA). After QC, 811 samples (319 females, 492 males, mean age 44.1 yrs) with overlapping genotype and 16S data were included in the microbiome GWAS.

The study was approved by the EUMC review board 2014-06-024 and KBSMC review board 2013-01-245. Written informed consent was obtained from all participants.

LLD (LifeLines-DEEP)

LLD is a subcohort of the prospective LifeLines cohort from the northern provinces of the Netherlands (Groningen, Drenthe and Friesland) and includes participant of Dutch ethnicity. Blood and fecal samples of LLD participants were collected between April and August 2013. Genotyping was performed using the Illumina ImmunoChip and Illumina Human CytoSNP-12 microarrays. Fecal DNA was extracted using the Qiagen AllPrep kit with a bead-beating step. Sequencing of the bacterial 16S gene, domain V4, was performed at the Broad Institute (Boston, USA) using the Illumina MiSeq platform. The overlap between samples with genotyping and microbial 16S sequencing data yielded 875 samples (504 females, 371 males, mean age 45.4(13.3) yrs) used for the microbiome GWAS analysis. Of these, 70 participants were PPI users and eight people used antibiotics in the six months prior to fecal collection. Each participant signed an informed consent form before participation in the cohort according to the UMCG Institutional Review Board (IRB; #M12.113965).

METSIM (METabolic Syndrome In Men)

The METSIM cohort is a longitudinal population-based cross-sectional cohort comprising 10,197 randomly selected non-diabetic Finnish men (aged 45 to 73 years) who were examined in 2005-2010. Genotyping was performed using the Illumina Omni ExpressExome microarray. Microbial DNA was extracted from frozen fecal samples using the PowerSoil DNA Isolation Kit (MO BIO Laboratories, Carlsbad, CA, USA) following the manufacturer's instructions. Amplification of the V4 hypervariable region of the 16S rRNA gene was done using the 515F and 806R primer and sequenced with the Illumina MiSeq platform at the University of California, Los Angeles. For the current microbiome GWAS study, we used a subset of the METSIM cohort consisting of 522 samples (mean age 61.91 (5.42) yrs) with overlapping genotyping and microbial 16S sequencing data. The study was approved by Ethics Committee of the Northern Savo Hospital District, Finland. Written informed consent was obtained from all participants.

MIBS (Maastricht Irritable Bowel Syndrome)

The MIBS cohort with biobank aims to identify subgroups of IBS according to phenotypical and genotypical characterization. At present, it includes 520 subjects with a clinical diagnosis of IBS according to the Rome III criteria (from primary-tertiary care) and 220 age- and gender-matched healthy controls. At baseline, all subjects completed an extensive questionnaire on

demographics, lifestyle factors, medical history and medication use, as well as a 14-day symptom dairy, the GSRS, HADS, STAI, SF-36 and a food frequency questionnaire. In addition, blood (serum, (platelet-poor) plasma, DNA), feces and exhaled air were collected. In subgroups, a rectal barostat and multisugar test for intestinal permeability was performed. All participants gave written informed consent. For the present microbiome GWAS study, only controls (N=80, mean age 48.7(18.2), 43% male) were included. Genotyping was performed using the Illumina ImmunoChip and Illumina Human CytoSNP-12 microarrays. Fecal DNA was extracted using the Qiagen AllPrep kit with bead-beating step. Sequencing of bacterial 16S gene, domain V4, was performed at the Broad Institute (Boston, USA) using the Illumina MiSeq platform. Each participant signed an informed consent form before participation in the cohort according to the Maastricht University Medical Center (MUMC+) IRB (#MEC 08-2.066.7/pl).

NGRC (NeurGenetics Research Consortium)

The NGRC is a collaborative study of gene-environment-microbiome interaction on Parkinson's disease. It is being conducted in the United States. A GWAS was conducted in 2009 on DNA from whole blood on the Illumina HumanOmni1-Quad_v1-0_B array[90]. Stool and metadata were collected from a subset of participants in 2014. Fecal DNA was extracted using the MoBio PowerMag Soil DNA Isolation Kit (Optimized for KingFisher). The 16S rRNA V4 amplicon was sequenced using the Illumina MiSeq platform[91]. For the microbiome GWAS study, only 133 control participants were used. These controls were free of neurodegenerative disease, had a mean age of 71.9 (7.5) years old and 58% were female.
The study was approved by the institutional review boards of the participating institutions: Albany Medical Center, Emory University; Kaiser Permanente Northwest Division, New York State Department of Health, Oregon Health & Sciences University (OHSU) and the Department of Veterans Affairs VA Puget Sound Health Care System (VAPSHCS). Written informed consent was obtained from all participants.

NTR (the Netherlands Twin Registry)

The NTR collects data and biological samples on Dutch multiples and their family members[92]. The NTR samples included in the microbiome GWAS were collected for two separate studies: the first focused on the association between obesity and the gut microbiome and the other collected samples from family members and spouses. Genotyping was performed on the

Affymetrix SNP 6.0, Affymetrix Axiom and Illumina GSA arrays. Fecal DNA was extracted using the Qiagen PowerSoil kit with the addition of the heating step of the Qiagen PowerFecal kit. The sequencing of the V4 domain of the 16S gene was performed using the Illumina MiSeq platform. DNA extractions and sequencing were performed at the Avera Institute for Human Genetics (Sioux Falls, SD, USA). One of each twin pair was randomly selected for inclusion in the GWAS analyses (156 twin pairs, 123 unrelated individuals, 279 individuals total, mean age 35.4(12), 29.8% male). Both MZ twins were included for the ICC calculations between MZ twin pairs for comparison with heritability estimates (156 twin pairs). None of the participants reported using antibiotics within six months of fecal collection.

The study was approved by Central Ethics Committee on Research involving human subjects of the VU University Medical Center, Amsterdam. Written informed consent was obtained from all participants.

PNP (Personalized Nutrition Project)

The PNP is a large-scale nutrition initiative in Israel that aims to help people make food choices that would normalize their blood glucose level and improve their health and well-being. The cohort has over 1,000 healthy individuals of Israeli ethnicity living in Israel and aged between 18 and 70 years. The cohort consists of self-reported Ashkenazi (n=508), North African (n=64), Middle Eastern (n=34), Sephardi (n=19), Yemenite (n=13) and ‘admixed/other’ (n=408) ancestries. The top two host genetic principal components (PCs) are strongly associated with self-reported ancestry ($P<10^{-32}$ for both PC1 and PC2, Kruskal-Wallis test). Participants were genotyped using Illumina OMNI-EXPRESS arrays. They also provided stool samples, which were collected using a swab or an OMNIGENE-GUT (OMR-200; DNA Genotek) stool collection kit. Metagenomic sequencing was performed on DNA extracted from the stool samples as was 16S rRNA profiling by sequencing the V3-V4 region. 481 individuals were included in the current study (mean age 43.7(13.1), 36.4% male).

The study was approved by Tel Aviv Sourasky Medical Center Institutional Review Board (IRB), approval numbers TLV-0658-12, TLV-0050-13 and TLV-0522-10; Kfar Shaul Hospital IRB, approval number 0-73; and Weizmann Institute of Science Bioethics and Embryonic Stem Cell Research oversight committee. Written informed consent was obtained from all participants.

PopCol (Population-based Colonoscopy)

PopCol is a cohort study in Stockholm, Sweden that includes a data-rich set of individuals with data available from bowel symptoms questionnaires, gastroenterology visits and biospecimens (genotype and 16S sequencing from blood and stool samples, respectively)[93,94]. Genotyping was carried out using the Illumina HumanOmniExpressExome-8v1 arrays at the SciLifeLab NGI facility in Uppsala, Sweden. Fecal DNA was extracted from samples kept at -80°C using Qiagen QIAamp DNA Stool Mini Kits and analyzed using 16S rRNA gene amplicon sequencing (in the V1-V2 hypervariable region). This was performed on the Illumina MiSeq platform at the Institute of Clinical Molecular Biology (IKMB) in Kiel, Germany. After data merging and QC, we used data from 134 individuals (83 females, 51 males, mean age 54.8(11.3) yrs) in the microbiome GWAS. Of these, six PopCol participants were PPI users and 12 used antibiotics. The study was approved by the local Committee of Research Ethics (Forskningskommitté Syd) at Karolinska Institutet, Stockholm, in November 2001. Written informed consent was obtained from all participants.

RS (Rotterdam Study III)

The RS is a prospective population-based cohort study established in 1990 to study determinants of disease and disability in Dutch adult/elderly individuals aged ≥40 years. The original design and updates of this study have been described in detail[95]. The RS consists of four sub-cohorts and comprises approximately 18,000 inhabitants of the Ommoord, a suburb of Rotterdam, the Netherlands. In the current microbiome GWAS, data from the Rotterdam Study III have been used. Genotyping was performed using the Illumina HumanHap 550K and 610K. The collection of fecal samples started in 2012 and includes 3,932 participants. Fecal DNA was extracted using DiaSorin Arrow DNA (Isogen Life Science, De Meern, the Netherlands) with a bead-beating step. Sequencing of bacterial 16S gene, domain V3-V4, was performed in the Laboratory of Human Genetics at Erasmus MC Rotterdam, using the Illumina MiSeq platform[89]. After stringent QC, the overlap between samples with genotyping and microbial 16S sequencing data yielded 1,220 samples (705 females, 515 males, mean age 57 (5.9) yrs) for use in the microbiome GWAS analysis. Of these, 260 participants used proton pump inhibitors and none used antibiotics in the six months before fecal collection.

The study was approved by the IRB (Medical Ethics Committee) of the Erasmus Medical Center and by the review board of the Netherlands Ministry of Health, Welfare and Sports. Written informed consent was obtained from all participants.

SHIP (Study of Health in Pomerania)

The SHIP is a prospective longitudinal population-based cohort study encompassing two independent cohorts: SHIP (N=4,308; baseline examinations 1997-2001) and SHIP-TREND (N=4,420; baseline examinations 2008-2012)[96]. Individuals were invited to the SHIP study center for computer-assisted personal interviews and extensive examinations. Follow-up investigations are scheduled at 5-year intervals and have already been performed three times for SHIP and once for SHIP-TREND. For the microbiome GWAS project, data from the second SHIP wave (SHIP-2, 2008-2012) and the initial recruitment phase of SHIP-TREND were used. Genotyping was performed using the Genome-Wide Human SNP Array 6.0 (Affymetrix, Santa Clara, CA, USA) or the Infinium HumanOmni2.5 BeadChip (Illumina, San Diego, CA, USA) for SHIP and SHIP-TREND, respectively. Isolation of fecal DNA was done using the PSP Spin Stool DNA Kit (Stratec Biomedical AG, Birkenfeld, Germany). Fecal microbiota composition was determined based on the V1-V2 regions of the 16S rRNA gene on a MiSeq platform (Illumina) at the Institute of Clinical Molecular Biology (Christian Albrechts University of Kiel, Germany), as described before[97]. After comprehensive QC, 1,901 datasets (1,043 females, 858 males, mean age 53.7(14.0) yrs) with overlapping genotype and microbiome data were included in the current study. Of these, 149 individuals used PPIs and 25 had antibiotics at the time of inclusion.

The study was approved by the medical ethics committee of the University of Greifswald. Written informed consent was obtained from all participants.

HCHS/SOL (Hispanic Community Health Study/Study of Latinos)

The HCHS/SOL is a prospective, population-based cohort study of 16,415 Hispanic/Latino adults (ages 18-74 years) who were selected using a two-stage probability sampling design from four US communities (Chicago, IL; Miami, FL; Bronx, NY; San Diego, CA)[98,99]. The genotyping of this cohort was performed with an Illumina custom array (15041502 B3), which consists of the Illumina Omni 2.5M array (HumanOmni2.5-8v1-1) plus ~150k custom SNPs, with the QC performed at the HCHS/SOL Genetic Analysis Center[100]. Stool samples were

collected in the HCHS/SOL Gut Origins of Latino Diabetes (GOLD) ancillary study, which enrolled participants from the HCHS/SOL approximately concurrently with the second visit for HCHS/SOL. Fecal DNA was extracted with the Qiagen MagAttract PowerSoil DNA kit with both chemical and physical (i.e. bead-beating) means to release DNA, as described in Marotz et al[101]. Sequencing of bacterial 16S gene, domain V4, was performed in Rob Knight's lab at the University of California San Diego (San Diego, CA, USA) using the Illumina MiSeq platform. After stringent QC, the overlap between genetically unrelated subjects with microbial 16S sequencing data yielded 1,097 samples (676 females, 421 males, mean age 57.2(10.9) yrs) used in the microbiome GWAS analysis. Of these, 341 used medication including PPI for indigestion, heartburn, or stomach problems and 321 used antibiotics in the six months before the fecal collection.

The study was approved by the Ethics and Institutional Review Boards of all institutions involved (Bronx Field Center – Albert Einstein School of Medicine; Chicago Field Center – University of Illinois Chicago; Miami Field Center – University of Miami; San Diego Field Center – San Diego State University). Written informed consent was obtained from all participants.

TwinsUK

TwinsUK is a population-based cohort established in 1992 to study the genetic and environmental basis of a range of complex diseases and conditions in adult/elderly twins from the UK[102]. Genotyping was performed using HumanHap610Q on 5,654 volunteers, followed by imputation. Fecal samples were collected between 2010 and 2016 for 1,793 of the genotyped twins. DNA was extracted using PowerSoil - htp DNA isolation kit and the V4 region of the 16s rRNA gene was sequenced using the Illumina MiSeq platform at the Department of Molecular Biology and Genetics, Cornell University, Ithaca, NY, USA. One twin out of each pair was randomly excluded from the population of 1,793 individuals, leaving 1,205 volunteers (1,101 females and 104 males, mean age 61.5(10.7) yrs) on which to conduct the microbiome GWAS analysis. Of these, 78 used PPIs and 62 had used antibiotics in the 6 months prior to sampling. The study was approved by the Cornell University IRB (Protocol ID 1108002388). Written informed consent was obtained from all participants.
