# Supplementary material for: The association between gut microbiota and accelerated aging and frailty: a Mendelian randomization study
Source: Aging Clin Exp Res. 2025 Mar 13;37(1):82. doi: 10.1007/s40520-025-02971-3 (PMC11903541; doi:10.1007/s40520-025-02971-3)
Supplement: Supplementary file 5 — Supplementary Material 5 [file 40520_2025_2971_MOESM5_ESM.pdf]

Author Contribution and Authorship Change Declaration

Manuscript Title: The association between gut microbiota and accelerated aging and frailty: a Mendelian randomization study

Manuscript ID: f4626327-955d-4245-be1c-66f3447486aa

Author list:

1. Zhiliang Yan Yanzhi liang 2025.01.21 ____________
2. Guoyu Guan Guoyu Guan 2025.01.21 ____________
3. Hanqi Jia Hanqi Jia 2025.01.21 ____________
4. Hanyu Li Hanyu Li 2025.01.21 ____________
5. Sangdan Zhuoga Sangdan Zhuoga 2025.01.21 ____________
6. Songbai Zheng Songbai zheng 2025.01.21 ____________ Corresponding author: songbai1009@163.com

Author contributions:

ZLY and SBZ participated in the design of the study and editing of the manuscript.

Material preparation was performed by GYG and HQJ.

Data collection and analysis were performed by ZLY and HQJ.

The first draft of the manuscript was written by ZLY, with all authors providing feedback on earlier versions.

ZSB was responsible for funding acquisition.

ZLY, HYL, and SDZ participated in the revision and proofreading of the manuscript.

Declarations: Zhiliang Yan, Guoyu Guan, Hanqi Jia, Hanyu Li, Sangdan Zhuoga and Songbai Zheng certify they have no relevant financial or non-financial interests in connection with the submitted article. The authors declare that they have no competing interests relevant to the content of this article and no conflicts of interest regarding its publication.

We sincerely apologize for any inconvenience caused by the authorship changes. We appreciate your understanding and patience, and once again, offer our apologies for any disruption this may have caused.
